# Eco-Epidemiological Profile and Molecular Characterization of Simian Foamy Virus in a Recently-Captured Invasive Population of *Leontopithecus chrysomelas* (Golden-Headed Lion Tamarin) in Rio de Janeiro, Brazil

**DOI:** 10.3390/v11100931

**Published:** 2019-10-10

**Authors:** Thamiris S. Miranda, Cláudia P. Muniz, Silvia B. Moreira, Marina G. Bueno, Maria Cecília M. Kierulff, Camila V. Molina, José L. Catão-Dias, Alcides Pissinatti, Marcelo A. Soares, André F. Santos

**Affiliations:** 1 Departamento de Genética, Universidade Federal do Rio de Janeiro, Rio de Janeiro 21941-617, Brazil; thamirismiranda02@gmail.com (T.S.M.); claudia.muniz16@gmail.com (C.P.M.); masoares@biologia.ufrj.br (M.A.S.); 2 Programa de Oncovirologia, Instituto Nacional de Câncer, Rio de Janeiro 20231-050, Brazil; 3 Centro de Primatologia do Rio de Janeiro, Instituto Estadual do Ambiente, Guapimirim 25948-395, Brazil; silviabm.inea@gmail.com (S.B.M.); alcidespissinatti@gmail.com (A.P.); 4 Instituto Pri-Matas para a Conservação da Biodiversidade, Belo Horizonte 31160-250, Brazil; buenomg@gmail.com (M.G.B.); ceciliakierulff@gmail.com (M.C.M.K.); camolina.vet@gmail.com (C.V.M.); 5 Laboratório de Virologia Comparada e Ambiental—LVCA, Instituto Oswaldo Cruz—IOC, Fundação Oswaldo Cruz-Fiocruz, Rio de Janeiro 21040-360, Brazil; 6 Instituto Nacional da Mata Atlântica—INMA, 29650,000, Santa Teresa, Espírito Santo 29932-540, Brazil; 7 Laboratório de Patologia Comparada de Animais Selvagens—LAPCOM, Departamento de Patologia, Faculdade de Medicina Veterinária e Zootecnia, Universidade de São Paulo, São Paulo 05508-270, Brazil; jlcataodias@gmail.com

**Keywords:** spumavirus, viral prevalence, epidemiology, Neotropical primates, free-living primates, Brazil

## Abstract

Simian foamy viruses (SFV) infect a wide range of Old World and Neotropical primates (NP). Unlike Old World primates, little is known about the diversity and prevalence of SFV in NP, mainly from a free-living population. Phylogenetic analyses have shown that SFV coevolved with their hosts. However, viral strains infecting *Leontopithecus chrysomelas* did not behave as expected for this hypothesis. The purpose of this study was to determine the eco-epidemiological profile and molecular characterization of SFV in a recently captured invasive population of *L. chrysomelas* located in Niteroi/RJ using buccal swab as an alternative collection method. A prevalence of 34.8% (32/92) and a mean viral load of 4.7 log copies of SFV/10^6^ cells were observed. With respect to time since capture, SFV prevalence was significantly higher in the group of animals sampled over 6 months after capture (55.2%) than in those more recently captured (25.4%) (*p* = 0.005). Infected solitary animals can contribute to SFV transmission between different groups in the population. SFV strains formed two distinct clades within the SFV infecting the Cebidae family. This is the first study to use buccal swabs as a tool to study SFV diversity and prevalence in a recently free-living NP population upon recent capture.

## 1. Introduction

Simian foamy viruses (SFV) are complex retroviruses that naturally infect a wide range of non-human primates, including Neotropical primates (NP) [1,2,3]. Phylogenetic analyses have indicated that SFV coevolved with nonhuman primates for at least 60 million years [4], contributing to the lack of pathogenicity observed in these animals [5]. In NP, the prevalence is generally higher in animals in captivity (45–51%) compared to animals in the wild (14–30%) [2,3]. Although SFV has been described in at least 23 species of NP [3], there are only five complete genomes sequenced [6,7,8,9], a small number considering the high diversity of NP, distributed in at least 176 species, 17–21 genera and three to five families according to distinct classification systems [10,11]. Although the first NP SFV has been identified over four decades ago in cell cultures of spider monkey (*Ateles sp*.) saliva [12], very little is known about the distribution, prevalence, and genetic variability of SFVs that infect this group, and most studies have been conducted with captive animals. Although there are two studies reporting SFV prevalence in free-ranging NP, they are restricted to a limited number of available specimens and species [2,3]. Therefore, the epidemiological profile of SFV for a given NP species and/or genera in the wild is at least an inaccurate estimate since it has never been evaluated at the population level.

*Leontopithecus chrysomelas* (golden-headed lion tamarin) is a small size NP belonging to the Cebidae family [13] categorized as *EN-Endangered* by The International Union for Conservation of Nature (IUCN) [14] and it is endemic in the south of Bahia state, Brazil [15]. However, a few *L. chrysomelas* individuals have been introduced into an urban Atlantic Forest fragment in Niterói city (Rio de Janeiro state, Brazil) by a private collector in the mid-90s, being considered as an exotic invasive species in this region [16]. This invasive population have had close contact with humans and domestic animals, entering at human houses and being fed by them, increasing the risk of virus transmission in both directions [17,18,19,20]. Moreover, the few introduced animals reproduced, becoming hundreds of animals, estimated in excess of 700 in late 2015 [21], and could be a threat to the local golden lion tamarin (*Leontopithecus rosalia*), an endangered species endemic to Rio de Janeiro state, with risks of disease transmission [14,15,16], competition by habitat and hybridization [16]. For those reasons, many *L. chrysomelas* family groups were captured as part of a conservation project to remove this introduced species, administered and conducted by the non-governmental organization Pri-Matas Institute since 2012. The captured animals were kept in quarantine at *Centro de Primatologia do Rio de Janeiro* (CPRJ; Guapimirim, RJ, Brazil) and between 2012 and 2013 some were translocated to an area in southern Bahia without *L. chrysomelas* and others groups were maintained in captivity [16].

Yet retroviruses have a close phylogenetic relationship with their hosts [22], the dynamics of infection can be influenced by ecological and behavioral factors, impacting their prevalence, virus–host interactions, within- and between-species transmission [23,24], and also transmission to the surrounding human population [25,26,27]. SFV transmission occurs mainly through bites and grooming [28]. Therefore, social behaviors that increase contact between individuals may potentiate the likelihood of SFV transmission, impacting SFV prevalence rates. In NP, there are different complex social and behavioral structures [29,30]; however, little is known about how these structures and anthropogenic actions impact the viral ecology of SFV in free-living animals. In a phylogenetic analysis early study by our group, the only SFV sequences obtained from a *L. chrysomelas* and a *L. rosalia* did not cluster to form a single clade for *Leontopithecus* [3]. Here, for the first time in NP, a large number of recently-captured *L. chrysomelas* specimens were analyzed, allowing us to deepen our knowledge on SFV prevalence, circulating viral genetic diversity, and how social behaviors and the environment may influence SFV transmission in this population.

## 2. Materials and Methods

### 2.1. Study Population and Ethics Statement

Buccal swab samples were collected from of 92 *L. chrysomelas* captured in Niterói city (Rio de Janeiro state, Brazil) in the period from December 2014 to September 2017. The captured specimens were distributed in 29 family groups and 4 were found to be solitary (captured alone). Each *L. chrysomelas* family group and solitary specimen was kept separated after their capture and have their material collected from two to fourteen months. Specimens were classified as two groups, those collected from two to six months after their capture and those collected from seven to fourteen months after their capture. The sexual maturity was classified according to size, dentition and weight of the specimens and these data, as well as gender, geographic location of capture and family groups were made available. The material and information were collected by the CPRJ and Pri-Matas veterinarians.

All procedures were conducted in full compliance with Federal permits issued by the Brazilian Ministry of the Environment (SISBIO 30939-12) and samples were collected following the national guidelines and provisions of IBAMA (Instituto Brasileiro do Meio Ambiente e dos Recursos Naturais Renováveis, Brazil; permanent license number 11375–1). The project was approved by the Ethics Committee on the Use of Animals (CEUA/CCS) of Universidade Federal do Rio de Janeiro, under the reference number 037-14.

### 2.2. Sample Collection, Processing and Confirmation of Genomic DNA Integrity

Buccal swabs were collected using sterile cotton swabs with a plastic shaft that was then placed into a sterile tube containing 500 μL of saline solution (0.9% NaCl), transported to the Genetics Department of Universidade Federal do Rio de Janeiro on ice and were stored at −80 °C until processing. Genomic DNA (gDNA) was extracted from buccal swab samples using the PureLink® Genomic DNA kit (ThermoFisher Scientific, Grand Island, NY, USA) according to the manufacturer’s specifications. After extraction, samples had their contaminants (PCR inhibitors) removed using the OneStep™ PCR Inhibitor Removal Kit (Zymo Research, Irvine, CA, USA). Shortly thereafter, samples were quantified using Nanodrop and stored at −20 °C. The integrity of the gDNA for PCR analysis was checked by PCR amplification of a mitochondrial constitutive gene (*cytB*) as previously described [1]. All DNA samples testing positive for *cytB* sequences were further considered suitable for SFV PCR detection.

### 2.3. Detection and Quantification of SFV

To detect NP SFV proviral DNA, we first performed a screening semi-nested PCR for short integrase sequences of 192 bp using generic primers and standard PCR conditions as previously described [1] as a diagnostic PCR test for NP SFV using 12 ng of buccal gDNA. In addition to the conventional diagnostic PCR for SFV detection, a real-time PCR assay was also performed to detect and quantify SFV viral copies in buccal swab samples, targeting a 124 bp region of the *pol* gene as previously described [31]. Primers and probes were designed using an alignment of available *pol* sequences from NP SFV, including representatives from all three NP families [1]. Briefly, one forward and one reverse primer were used (QSIP4Nmod (for) 5′-TGC ATT CCG ATC AAG GAT CAG C-3′ and QSIR1Nmod2 (rev) 5′- TTC CTT TCC ACY WTY CCA CTA CT-3′), with the probe DIAPR2 5′-FAM- TGG GGI TGG TAA GGA GTA CTG WAT TCC A-SpC6-3′. Following a 10 min incubation at 95 °C to activate Taq polymerase, a three-step PCR was performed at 95 °C for 15 sec, 50 °C for 15 sec, and 62 °C for 15 sec for 55 cycles using the 7500 Real-Time PCR platform (Applied Biosystems, Foster City, CA, USA). The sensitivity of the assay was 100 copies of SFV/reaction, as determined in [31].

To normalize the amount of diploid cells per reaction, the mean number of housekeeping gene ribonuclease P/MRP 30 kDa subunit (*RPP30*) copies of five *L. chrysomelas* swab samples (284 copies/ng) was used, as described previously [31]. Thus, since each cell has two copies of the RPP30 gene, the mean used was 142 cells/ng of DNA in buccal swab samples of *L. chrysomelas*.

### 2.4. Amplification of a Larger SFV Fragment from the Cebidae Family

For the positive samples in at least one of the SFV detection tests (diagnostic PCR or real-time PCR), one additional PCR was carried out to amplify a larger SFV subgenomic region for phylogenetic analysis. Despite the controversy about the number of NP families, the *Leontopithecus* genera was classified as part of the Cebidae family in the present report [11]. Primers were then designed using a conserved region of *pol* in an alignment of two SFV complete genomes representing the Cebidae family available at GenBank from marmoset and yellow-breasted capuchin (accession numbers GU356395 and KP143760, respectively) [7,9] and *pol* sequences generated by the diagnostic PCR test in this study as describe above. Briefly, the new nested PCR was performed using primers: (1° Round: *pol*5474 5′ GCCAAACATGAGAAAGGATG 3′ and *pol*5960 5′ TACCACTTTGTAGGTCTTCC 3′ with annealing temperature of 53.4 °C) and (2° Round: *pol*5500 5′ GTCATATCCGTAYGTGCAAAC 3′ and *pol*5878 5′CTTTGGGGGTGGTAAGG 3′ with annealing temperature of 56 °C), amplifying a 378 bp fragment.

In addition, primer combinations were tested in the 2° round above to analyze the amplification efficiency of the viral fragments. The *pol*5474 *and pol*5878 primers (annealing temperature of 54 °C), and *pol*5500 with *pol*5960 (annealing temperature of 56 °C) were combined, amplifying fragments of 404 bp and 460 bp, respectively. PCR were performed with an initial temperature of 94 °C, followed by 35 cycles of 94 °C for 30 seconds, primer annealing at temperatures varying according to the combination of primers for 30 sec and 72 °C for 90 sec. Products were sequenced by the Sanger method using and ABI 3130 XL automatic sequencer (Applied Biosystems) and primers of the second round.

### 2.5. Sequence Analysis

Generated sequences were submitted to the BLASTn tool (http://blast.ncbi.nlm.nih.gov) for similarity analysis with SFV sequences deposited at Genbank. SFV sequences were edited using the SeqMan program v.7.0 (DNASTAR, Madison, WI, USA) and aligned with NP SFV reference sequences deposited in GenBank using the BioEdit Sequence Alignment Editor v.7.0.4 [32]. From the alignment, a phylogenetic tree was generated with Mega7 [33], using the maximum likelihood method and the Tamura 3-parameter correction model with discrete gamma rate variation. The bootstrap method was used with 1000 replicates to estimate the reliability of the phylogenetic clusters. Values above 70% were considered significant [3]. A similarity analysis between SFVlcm strains in the *pol* sequence was conducted using the Maximum Composite Likelihood model. The analysis involved 13 nucleotide sequences. All positions containing gaps and missing data were stripped. There was a total of 263 nucleotide positions in the final dataset. Evolutionary analyses were conducted in MEGA7. The geographical distribution of the *L. chrysomelas* family groups was plotted using the coordinates of family groups using program RStudio [34] using package Leaflet OpenStreetMap^®^ contributors under license Open Data Commons Open Database License (ODbL).

### 2.6. Statistical Analyses

To understand better the epidemiology of SFV in *L. chrysomelas,* differences in SFV prevalence were evaluated with Chi Square trend analysis for the following categories: males and females; and infants, juveniles and adults. We also divided the familiar groups according to the time elapsed between capture and collection: one group between 1 and 6 months of captivity time (*n* = 63) and another group between 7 and 14 months in captivity (*n* = 29). After logarithmic transformation of SFV viral load data, *T*-tests were performed to test for associations between viral loads and all the characteristics mentioned above. For low sample numbers (*n* < 20), the Fisher Exact Test was used to evaluate epidemiological prevalence between groups with different captivity times and the efficiency of amplification by the diagnostic PCR between the two strains of SFVlcm described in this study. For all these tests, *p*-values ≤ 0.05 were considered significant.

### 2.7. Data Availability

All SFV sequences generated herein have been deposited at GenBank with the accession numbers MN178627 to MN178637.

## 3. Results

### 3.1. Population Profile

Samples of 29 golden-headed lion tamarin family groups were collected in this study. The mean number of specimens captured was eight per group, ranging from three to 12, while the mean number of specimens collected was three by group, ranging from one to seven animals. Of the 92 animals collected at CPRJ, we found a higher proportion of males (56.5%) than females (43.5%) (Table 1). With respect to sexual maturity, the specimens analyzed were constituted largely by adults (*n* = 45; 49%), followed by juveniles (*n* = 32; 35%) and infants (*n* = 15; 16%). All gDNA samples extracted from buccal swabs were positive for the constitutive mitochondrial *cytB* gene, and therefore were considered suitable to the molecular tests for SFV detection and quantification.

### 3.2. SFV Molecular Detection and Quantification

A sample was considered positive for SFV infection when it tested positive in either one of the two molecular tests used (conventional diagnostic PCR and/or qPCR). Using this criterion, 15 samples (16.3%) were positive by diagnostic PCR and 28 (30.4%) were positive by qPCR (Table 1). When comparing the two assays, the results were 70% concordant. Of the discordant results, qPCR was more sensitive (17%) than the conventional diagnostic PCR (4%) (*p* = 0.006). Thus, 32/92 (34.8%) of the animals were considered infected with SFV (Table 1). Females and males presented similar SFV prevalence (40%) and (30.7%), respectively (*p* = 0.483). Regarding sexual maturity, no statistical difference was observed in the prevalence of SFV infection between different groups (*p* = 0.502) (Table 1). By grouping infants with juvenile specimens and comparing with adults, the SFV prevalence between immature and mature animals was very similar, 17/47 (36.2%) and 15/45 (33.3%), respectively (*p* = 0.946).

We sought to address whether the low sensitivity of the conventional diagnostic PCR was related to the lower number of SFV DNA copies of the negative samples for diagnostic PCR, but positive for qPCR, but there was no correlation between those conditions (*p* = 0.175). Among the 28 samples that had detectable SFV DNA VL, after normalization with the mean RPP30 copies in buccal swab cells, the mean VL was 4.7 log copies of SFV/10^6^ cells, ranging from 3.47 to 5.98 log copies/10^6^ cells. No differences were found between oral SFV DNA VL of males (*n* = 14) and females (*n* = 14) (mean of 4.7 log and 4.8 log copies/10^6^ cells, respectively; *p* = 0.735). Regarding sexual maturity, the mean DNA VL were also similar between the different groups: infants (*n* = 4), juveniles (*n* = 10) and adults (*n* = 14) with means of 4.7, 4.6 and 4.7 log copies/10^6^ cells, respectively (*p* = 0.254).

With respect to time in captivity, SFV prevalence was lower in animals kept in captivity within 1–6 months (25.4%; 16/63) than in animals that stay in captivity more than seven months (55.2%; 16/29) (*p* = 0.005) (Table 2). The SFV prevalence among females also differed in the two groups with 32% (9/28) in the former group and 66.6% (8/12) in the latter (*p* = 0.042). The same was observed to male infections, with 20% (7/35) in the shorter captivity group and 47.1% (8/17) in the longer captivity group (*p* = 0.043). The SFV prevalence was also higher in the longer captivity group among all different age groups. However, only in juveniles had SFV prevalence reached a borderline statistical significance (*p* = 0.055; Table 2).

### 3.3. Phylogenetic Analysis and Similarity of SFV from L. Chrysomelas

**Figure 1:**
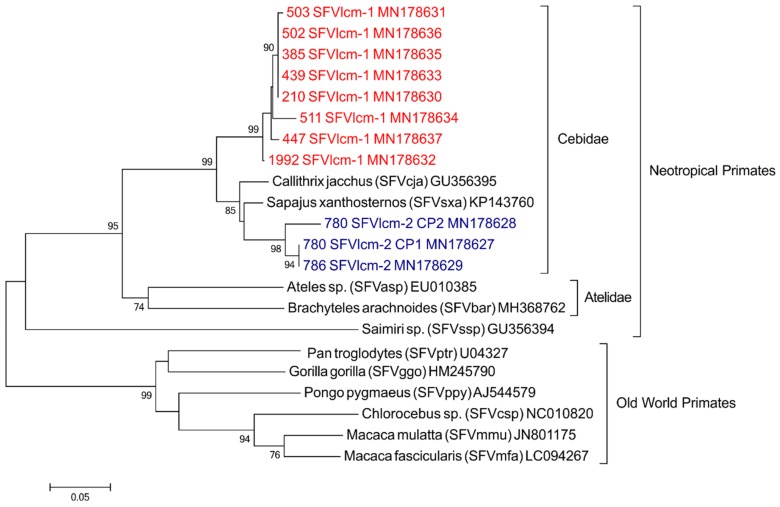
Platyrrhini SFV: phylogeny tree inferred using maximum likelihood analysis with a fragment of viral polymerase (360 bp). New sequences generated in the current study are marked in red (cluster SFVlcm-1) and in blue (cluster SFVlcm-2), all deposited at GenBank under the accession numbers MN178627 to MN178637. Bootstrap support was determined using 1000 nonparametric resampling replicates and values ≥ 70% are provided at nodes.

To perform a phylogenetic analysis and to infer the evolutionary history of the SFV that infect this population of *L. chrysomelas*, it was necessary to amplify larger PCR fragments. Of the 32 SFV previously positive samples, three samples amplified a 378 bp fragment with the 5500 and 5878 primer combination (see Methods); another six samples amplified a 404 bp fragment with the 5474 and 5878 primer combination and two additional samples amplified a 460 bp fragment with the 5500 and 5960 combination of primers tested in the second round PCR. Only two samples amplified for two different primer combinations (5500 and 5878; 5474 and 5878). However, for one of the samples (specimen 780), the two primer combinations amplified two distinct variants (Figure 1). In total, 10 animals amplified for larger region of *pol*. Due to a short sequence overlap of our generated sequences with the SFV *pol* sequences available from Genbank, the phylogenetic analysis was limited only to the five complete NP SFV genomes available in the literature. The analysis suggests there are two distinct lineages of SFV co-circulating in the population of *L. chrysomelas* analyzed; a major lineage, herein named SFVlcm-1 (described in red; Figure 1), formed a single clade that branches out of the other SFVs infecting the Cebidae family, and another lineage (SFVlcm-2; described in blue), formed a clade with SFV infecting *Sapajus xanthosternos* and *Callithrix jacchus* (Figure 1). As expected, both strains clustered within the viruses infecting the Cebidae family. When analyzing the PCR amplification efficiency of NP SFV between the two strains found, we observe that among SFVlcm-1 strain only 25% (2/8) amplified by the conventional diagnostic PCR, whereas SFVlcm-2 strain had 100% (2/2) of the strain PCR-amplified (*p* = 0.520).

The pairwise distance analysis showed that the sequences within each strain are similar to each other, with an average divergence of 1% within strain 1 and of 2.6% within strain 2. When comparing SFVlcm-1 to -2, the mean divergence between them was 11%, higher than when compared strain 1 to sequences of other representatives of the Cebidae family, 8.5% and 8.8% for *Sapajus* and *Callithrix*, respectively (Table 3).

### 3.4. Evaluation of SFV Transmission among Groups of L. chrysomelas

**Figure 2:**
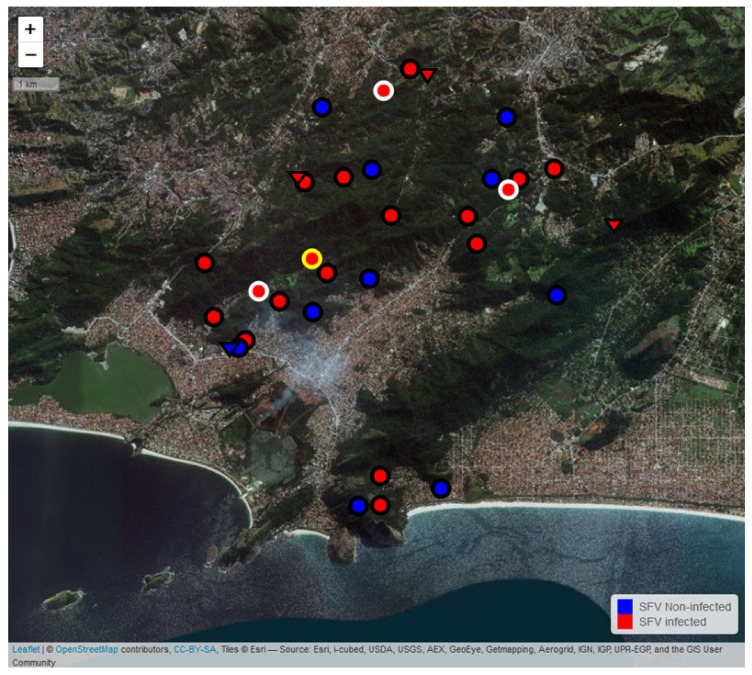
Eco-epidemiology of SFV in the *L. chrysomelas* family groups in the city of Niteroi/RJ. The location of each *L. chrysomelas* family group is represented by circles and of solitary animals by triangle. The red color represents infected animals or groups (when at least one animal is infected in the group), while the blue color represents the uninfected animals measured by conventional diagnostic PCR and/or quantitative PCR. Gray halos around the circles depict the presence of the SFVlcm-1 strain, while the yellow halo represents the SFVlcm-2 strain. The absence of halos indicates lack of amplification of the larger pol fragment, not allowing the classification in SFVlcm-1 or 2.

To investigate the eco-epidemiological profile of the SFV infection among the *L. chrysomelas* groups, a map was plotted using the GPS coordinates obtained during specimens’ captures in the forest area of Niteroi city to analyze SFV distribution (Figure 2). The viral distribution among the family groups was widely disseminated in the population. The SFVlcm-1 strain was present in three spatially separated groups and the SFVlcm-2 strain was limited to a single group. All groups belonged to the central forest area (Figure 2).

Interestingly, we observed that of the four solitary animals, three were infected by SFV (Figure 2). Three were males and one was female, with an SFV prevalence of SFV of 67% (2/3) and 100% (1/1), respectively. All the solitary animals were adults. These data suggest that errant males and females can contribute to the spread of SFV infection within this free-ranging primate population.

## 4. Discussion

The study of SFV in NP can be specially challenging due to the difficult access to free-living primates and limited volumes of blood that can be collected, since many specimens have a small size [29]. Moreover, of the 176 species of NP that circulate in Brazil, many are threatened to extinction [35]. To detect the SFV provirus, a high mass of genomic DNA (250–500 ng) is necessary from peripheral blood mononuclear cells [1], since blood cells are a recognized site of foamy virus latency [36]. Therefore, the use of buccal swabs is an important tool for SFV detection, since it preserves the animal’s health and provides a higher viral load since the oral mucosa is a major SFV replication site [28,31]. Thus, alternative stress-relieving methods, such as buccal swab, are attractive sample sources for the study of SFV, mainly in small primates threatened to extinction.

In the more recently-captured subgroup studied here, an SFV prevalence of 25.4% was observed, similar to the one found in previous studies with free-living primates (14–30%) [2]. However, the SFV prevalence of the subgroup with longer time in captivity was much higher (55.2%), in agreement to the observed in captive Peruvian and Brazilian primates (45–51%) [2,3]. This increase in SFV prevalence among animals kept in captivity for longer occurred for both sexes and mainly among juveniles. Although this population of *L. chrysomelas* lived in a restricted fragment of Atlantic forest, favoring the contact between groups, the captivity environment clearly contributed to increased transmission of SFV. It is known that an environment that does not promote the welfare and interest of the animal can generate stress, which can be reflected in behavioral changes such as increased aggressiveness [37], but likely also in the susceptibility to infectious agents. Transmission of SFV can happen through blood transfusion [38], maternal milk [39] and mainly by biting and grooming [5]. Thus, stressful environments can collaborate for a higher dissemination of SFV between captive animals.

The area where these animals have occupied is very fragmented (Figure 2), and some areas are very close to urban areas, where many of these animals were seen close to household waste to feed [18]. The proximity between non-human primates and humans can contribute to a risk of SFV zoonotic transmission to the latter [25,26,27]. Although until now SFV is not known to cause disease in its natural hosts [5], the association of SFV infection with mild anemia was observed in humans [40]. Little yet is known about the transmissibility of SFV from NP to humans and their consequences, but, previous work by our group has shown prevalence rates of SFV zoonotic transmission to primate handlers using serological assays [25]. We are currently working with primate handler samples to deepen our knowledge of NP SFV zoonotic transmission.

When conventional diagnostic PCR was standardized, there were only three complete NP SFV genomes available at Genbank, and the sensitivity of the assay was measured at 100% in seven NP genera studied (*Cebus, Alouatta, Callithrix, Aotus, Ateles, Saimiri, Cacajao* and *Pithecia*) [1]. However, sensitivity drops too much for detecting SFV from other genera such as *Leontopithecus*. Therefore, a quantitative PCR was developed using all available NP SFV *pol* sequences as references for degenerate primer design that amplifies a smaller and more conserved *pol* gene region. This assay was able to detect SFV in two additional species of *Leontopithecus*, and in one species each of *Callimico* and *Saguinus* previously found to be SFV-negative using the conventional diagnostic PCR assay [31]. As demonstrated previously [31], the qPCR was shown to be more sensitive than the conventional diagnostic PCR for detection of NP SFV, When correlating the number of SFV copies with the sensitivity of conventional diagnostic PCR, similar to what has been observed for feline FV [41], no association was found. These results suggest that the false negatives in the conventional PCR may be due to a high genetic heterogeneity of NP SFV sequences at primer locations determined previously to be 41% in the virus *pol* region [1].

SFV DNA VL comprises both the integrated virus (provirus) and the genomic DNA of the virus particle, since SFV can produce both DNA and RNA particles [5]. Little is known about the standards of the DNA VL in the oral mucosa of NP. A recent study [31] found a mean viral load of 4.7 log SFV copies/10^6^ cells among 23 NP specimens of 12 different species in captivity, including four *L. chrysomelas* specimens [31], similar to that has been found in this study. However, when comparing the VL of only four *L. chrysomelas* and one *L. rosalia* quantifiable for SFV of the previous study (range 2.9–7.3 log SFV copies/10^6^ cells), the variation was much higher than the one observed in this study (standard deviation 0.62), which can be explained by differences in sample size. In addition, no association was found regarding the viral load and the sex of the animal, also as observed in the previous study. Finally, also as in the previous study [31], we could not observe any age-related viral load trends in buccal swab samples. These results differ from those reported for rhesus macaques, in which viral load increases with age in the oral cavity of the animals [36]. However, it should be noted that we quantified VL DNA instead of VL RNA, as reported in the rhesus study, and that may explain such lack of correlation observed here. Another important issue is that Liu et al. [24] tested 173 fecal samples from wild chimpanzees (including 87 SFVcpz RNA-positive samples), and none of them detected viral DNA. DNA genome particle production may not reflect replication *in vivo*, and may represent an *in vitro* artifact when using tumor cells with high dNTP levels. Thus, it is unclear whether the viral detection tests (conventional diagnostic PCR and quantitative PCR) in this study are also detecting viral DNA but only proviral DNA.

When comparing the impact of different demographic factors on SFV prevalence, either in the population or in subgroups according to the captivity time, we observed that, as described by others [1,2,3], the animal sex does not seem to influence SFV acquisition. Yet it has been reported that SFV prevalence increases with age [3,39], no such correlation was observed here. This homogenization in the prevalence between the age groups can be explained, at least in part, by the social behavior of *Leontopithecus*. Group members do social grooming, all members of the family groups help to carry the offspring of the alpha couple and in nature (or captivity), all individuals sleep together, often in the hollows of trees [42], intensifying the contact between them and consequently the chance of SFV transmission. Another interesting ecological characteristic of many NP like *L. chrysomelas,* upon reaching maturity and especially males, is to leave their groups to form a new family group to avoid consanguinity [42]. Interestingly, of the four solitary animals, three were infected, even though they were kept isolated after months at CPRJ since their capture in the wild, showing that these animals may contribute to the dissemination of SFV in the population by entering into existing groups or forming new groups. Our results demonstrate for the first time a new SFV transmission dynamics in primates, on an ecological scale, highlighting the importance of molecular and ecological virology studies in free-living primates.

The use of primers for PCR amplification of larger fragments of the SFV LTR-*gag* region and the *pol* region from previous studies showed a low efficiency to amplify SFV from *L. chrysomelas* [1,3], indicating that SFVlcm can harbor a high nucleotide heterogeneity, at least in the region of primer annealing. To amplify larger DNA fragments, we developed new PCR primers only using sequences of representative SFV genomes infecting primates of Cebidae family to increase specificity. However, the new PCR amplified only 24% of samples diagnosed as SFV-positive, suggesting that there may be more variants circulating in the population, requiring more sensitive techniques, such as shotgun next-generation sequencing, to amplify the complete genomes of these viruses [6].

Phylogenetic analysis showed that, unlike a previous study [3] where a SFV sequence of *L. chrysomelas* clustered with SFV infecting Pitheciidae family members, all SFV sequences from *L. chrysomelas* here in generated grouped into the Cebidae family, which is expected according to the co-speciation hypothesis [4]. However, two distinct lineages of SFVlcm were observed. The most frequent, which formed a separate clade, was named SFVlcm-1, while the other, SFVlcm-2, formed a clade with SFVsxa and SFVcja, infecting *Sapajus xanthosternos* and *Callithrix jacchus*, respectively (Figure 1). The nucleotide divergence between the two strains was 11%, although this refers to a small fragment of the viral *pol* gene, which is conserved among SFVs. Since an earlier study has reported the occurrence of cross-species transmission between *L. chrysomelas*, *Sapajus xanthosthernos* and Pitheciidae species [3], cross-species transmission between species may have occurred in the Atlantic forest fragment where *L. chrysomelas* lives, where there are reports of other PN species, such as *Callithrix jacchus*. Another possibility could indicate that these variants came from cross-species transmission events prior to the arrival of the specimens in Niteroi/RJ, since in the endemic area of Bahia there are also other species of primates, as black-tufted marmoset (*Callithrix penicillata*) and yellow-breasted capuchin monkey (*Sapajus xanthosthernos*). However, as the phylogenetic inference of the two lineages was limited to only five complete NP SFV genomes available in the literature, of only two NP families, and was based on short *pol* sequences, we cannot assess whether the SFVlcm-2 clade was derived from a recombination event between SFVlcm-1 and an SFV from another species, as it has been already described in SFV-Infected Old World monkeys [43]. These alternative scenarios turn the understanding of the complete evolutionary history of the SFV infecting *L. chrysomelas* a difficult task at the moment. This issue will only be clarified with the amplification of larger SFV sequences or complete genomes from *L. chrysomelas* derived from the native population of Bahia and other NP SFV representatives.

In conclusion, we have demonstrated here for the first time an increase in the SFV prevalence of recently-captive *L. chrysomelas*, including the characterization of two novel SFV strains, SFVlcm-1 and -2, by using oral swab as an efficient alternative non-invasive method. We also present new ecological dynamics of SFV transmission from infected solitary animals that dispersed to form new groups or joined existing groups. Further studies are needed to fully characterize the SFV variants in this species, only preliminarily described here, which will improve our understanding of retroviral infections in the Platyrrhini parvorder, covering all primates of the Americas.

## Figures and Tables

**Table 1:** Comparison of simian foamy virus (SFV) prevalence estimates by quantitative PCR (qPCR) and conventional PCR (cPCR) in relation to sex and the sexual maturity of *L. chrysomelas.*

Characteristic	N (%)	qPCR+/cPCR+	qPCR+/cPCR-	qPCR-/cPCR+	qPCR-/cPCR-	SFV Prevalence (%)
**Total**	92	11 (12%)	17 (18.5%)	4 (4,3%)	73 (58%)	32/92 (34.8%)
**Sex**						
Male	52 (56.5%)	6 (11.5%)	8 (15.3%)	2 (4%)	36 (66.2%)	16/61 (30.7%)
Female	40 (43.5%)	5 (12.5%)	9 (22.5%)	2 (5%)	24 (60%)	16/40 (40%)
**Sexual maturation**						
Infants	15 (16%)	1 (6.7%)	3 (20%)	2 (13.3%)	9 (60%)	6/15 (40%)
Juveniles	32 (35%)	3 (9.4%)	7 (22%)	1 (3%)	21 (65.6%)	11/32 (34.4%)
Adults	45 (49%)	7 (15.5%)	7 (15.5%)	1 (2.2%)	30 (66.7%)	15/45 (33.3%)

**Table 2:** Comparison of SFV prevalence in relation to sex and sexual maturity of groups classified according to captivity time at Centro de Primatologia do Rio de Janeiro (CPRJ).

	1–6 Months	7–14 Months	*p*-Value
**Gender**			
Male	7/35 (20%)	8/17 (47%)	0.043
Female	9/28 (32%)	8/12 (67%)	0.042
**Sexual Maturity**			
Infants	4/12 (33%)	2/3 (67%)	0.525
Juveniles	4/19 (21%)	7/13 (54%)	0.055
Adults	8/32 (25%)	7/13 (54%)	0.062
**Total**	16/63(25%)	16/29 (55%)	0.005

**Table 3:** Evolutionary divergence estimates between SFV sequences from Cebidae.

	1	2	3	4	5	6	7	8	9	10	11	12
1.210 SFVlcm-1												
2.385 SFVlcm-1	0.000											
3.439 SFVlcm-1	0.000	0.000										
4.447 SFVlcm-1	0.012	0.012	0.012									
5.502 SFVlcm-1	0.000	0.000	0.000	0.012								
6.503 SFVlcm-1	0.004	0.004	0.004	0.015	0.004							
7.511 SFVlcm-1	0.023	0.023	0.023	0.027	0.023	0.027						
8.1992 SFVlcm-1	0.011	0.011	0.011	0.015	0.011	0.015	0.035					
9.780 SFVlcm-2_CP1	0.116	0.116	0.116	0.107	0.116	0.121	0.106	0.103				
10.780 SFVlcm-2_CP2	0.125	0.125	0.125	0.116	0.125	0.130	0.120	0.121	0.039			
11.786 SFVlcm-2	0.116	0.116	0.116	0.107	0.116	0.121	0.106	0.103	0.000	0.039		
12.SFVsxa	0.090	0.090	0.090	0.077	0.090	0.094	0.076	0.077	0.051	0.081	0.051	
13.SFVcja	0.090	0.090	0.090	0.090	0.090	0.094	0.085	0.081	0.064	0.094	0.064	0.039

The number of nucleotide substitutions per site between sequences is shown. Standard error estimates are shown above the diagonal. Analyses were conducted using the Maximum Composite Likelihood model. The analysis involved 13 nucleotide sequences. Codon positions included were 1st + 2nd + 3rd. All positions containing gaps and missing data were stripped. There were a total of 263 nucleotide positions in the final dataset. Evolutionary analyses were conducted in MEGA7. The colors represent the viral strains: in red SFVlcm -1; blue SFVlcm-2 and gray the complete genomes of SFVsxa and SFVcja.
